# A new approach to assessing the space use behavior of macroinvertebrates by automated video tracking

**DOI:** 10.1002/ece3.7129

**Published:** 2021-03-13

**Authors:** Milad Shokri, Francesco Cozzoli, Mario Ciotti, Vojsava Gjoni, Vanessa Marrocco, Fabio Vignes, Alberto Basset

**Affiliations:** ^1^ Laboratory of Ecology Department of Biological and Environmental Sciences and Technologies University of the Salento Lecce Italy; ^2^ Research Institute on Terrestrial Ecosystems (IRET) ‐ National Research Council of Italy (CNR) via Salaria Roma Italy

**Keywords:** body size, Ethovision, giving‐up time, macroinvertebrates, Noldus, space use

## Abstract

1. Individual space and resource use are central issues in ecology and conservation. Recent technological advances such as automated tracking techniques are boosting ecological research in this field. However, the development of a robust method to track space and resource use is still challenging for at least one important ecosystem component: motile aquatic macroinvertebrates. The challenges are mostly related to the small body size and rapid movement of many macroinvertebrate species and to light scattering and wave signal interference in aquatic habitats.
2. We developed a video tracking method designed to reliably assess space use behavior among individual aquatic macroinvertebrates under laboratory (microcosm) conditions. The approach involves the use of experimental apparatus integrating a near infrared backlight source, a Plexiglas multi‐patch maze, multiple infrared cameras, and automated video analysis. It allows detection of the position of fast‐moving (~ 3 cm/s) and translucent individuals of small size (~ 5 mm in length, ~1 mg in dry weight) on simulated resource patches distributed over an experimental microcosm (0.08 m^2^).
3. To illustrate the adequacy of the proposed method, we present a case study regarding the size dependency of space use behavior in the model organism *Gammarus insensibilis*, focusing on individual patch selection, giving‐up times, and cumulative space used.
4. In the case study, primary data were collected on individual body size and individual locomotory behavior, for example, mean speed, acceleration, and step length. Individual entrance and departure times were recorded for each simulated resource patch in the experimental maze. Individual giving‐up times were found to be characterized by negative size dependency, with patch departure occurring sooner in larger individuals than smaller ones, and individual cumulative space used (treated as the overall surface area of resource patches that individuals visited) was found to scale positively with body size.
5. This approach to studying space use behavior can deepen our understanding of species coexistence, yielding insights into mechanistic models on larger spatial scales, for example, home range, with implications for ecological and evolutionary processes, as well as for the management and conservation of populations and ecosystems. Despite being specifically developed for aquatic macroinvertebrates, this method can also be applied to other small aquatic organisms such as juvenile fish and amphibians.

## Graphical abstract

We developed a video tracking method designed to reliably assess space use behavior among individual aquatic macroinvertebrates.To illustrate the utility of the proposed method, we present a case study regarding the size dependency of space and resource use behavior in Gammarus insensibilis, focusing on individual's patch selection, giving‐up time, and cumulative space used .We observed the negative size dependency of individual giving‐up time, showing that patch departure occurred sooner in larger individuals than smaller ones and that individual cumulative space used scaled positively with body size.

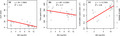

## 1 INTRODUCTION

How an organism uses space and invests its time in a heterogeneous environment influences its evolutionary success and hence its population dynamics (Bogdziewicz et al., 2016; Cayuela et al., 2017). Space use behavior is a key phenotypic trait (Avgar et al., 2015) affecting various components of individual fitness such as access to trophic resources and energy gain (Koy & Plotnick, 2007; Smith et al., 1983), management of predation risk (Beckerman et al., 2010; Makin & Kotler, 2019), and reproductive success (Getz et al., 2005). Moreover, the spatial dimensions of individual niches (rather than trophic dimensions) have been proposed as major drivers of interspecific coexistence (Melbinger & Vergassola, 2015).

Recent theoretical and experimental efforts have highlighted the relevance of various individual movement modes, for example, searching (Auger‐Méthé et al., 2015; Chakravarty et al., 2019), patch use, and giving‐up behaviors (Brown, 1988; Charnov, 1976; Davidson & Hady, 2019). Studies of animal movement have become more frequent in recent years, emphasizing the conceptual significance of space use behavior (Barela et al., 2020; Nathan et al., 2008; Potts et al., 2014). Nevertheless, gaining insight into the motivations and limitations of individual behavior is still a major challenge in empirical studies (Froy et al., 2018), with implications for theoretical development (Stevens, 2010).

So far, studies of space use behavior have focused mostly on larger animals living in environments where their movement can be followed relatively easily by radio and global positioning (*GPS*) collars (Cagnacci et al., 2011; Mysterud et al., 2011), passive radio frequency identification (*RFID*) and transponders (*PIT tags*) (Brodersen et al., 2008; Chapman et al., 2011). Examples include migrating birds, mice and other rodents, wandering whales, and fish (Edwards et al., 2007; Gurarie et al., 2009; Humphries et al., 2012; Reubens et al., 2019). Coupled with improving technology, surveys of smaller animals (mostly terrestrial) have also recently become more numerous (Barnes et al., 2015; Robinson et al., 2009; de la Rosa, 2019).

In contrast, little is known about the space and resource use dynamics of aquatic invertebrates (*but see “*Kölzsch et al. (2015); Cloyed and Dell (2019); Cloyed et al. (2019)") and additional technical challenges need to be overcome before studying these sensitive animals and subjecting them to experimental manipulation. Mark and recapture is used in space use behavior studies, for example, Davy‐Bowker (2002), but it is widely acknowledged that marking or tagging can lead to deviations from aquatic organisms’ normal behavior (Hagler & Jackson, 2001). Potential negative effects include changes in activity level and swimming performance, reduced feeding and growth, and lower survival rates (Cooke et al., 2011; Jepsen et al., 2015). Major negative effects are indeed reported in 17% of the peer‐reviewed literature (Lameris & Kleyheeg, 2017). It should also be considered that most studies of macroinvertebrate movement are performed at the population level (Holyoak et al., 2008), despite space use behavior varying among individuals, especially when strong phenotypic differences exist (Nonacs, 2001; Roche et al., 2016). This variation has the potential to drive patch selection at the population level, with cascade effects on community and ecosystem functioning (Post et al., 2008).

In this study, we propose a new method, derived from the experimental approaches of Mancinelli (2010); Dell et al. (2014); Augusiak and Van den Brink (2015); Barnes et al. (2015); Cloyed and Dell (2019); Cloyed et al. (2019), in order to track the movements of small aquatic macroinvertebrates. This method provides high‐throughput and accurate spatial data which can be assessed with reference to theoretical and conceptual frameworks. The experimental tracking system was created ad hoc by Noldus Information Technology (https://www.noldus.com) and consists of a near infrared backlight source (*NIR*) with a rigid infrared‐sensitive camera positioned directly above it. The combination of near infrared backlight and an infrared‐sensitive camera makes it possible to detect very small and fast‐moving animals in the absence of visible light.

To illustrate the potential of this equipment and method, we present a case study using a set of measures of variation of individual space‐use behavior along a gradient of individual body size. We used an indoor microcosm system under controlled experimental conditions in order to exclude factors that could potentially interfere with the normal behavior of the animals, for example, temperature (Lagerspetz & Vainio, 2006), resource quality (Fernandez et al., 2019), and circadian rhythms (Golet et al., 2006). Our principle focus was the size gradient, since body size affects virtually all aspects of individual physiology and foraging ability throughout the hierarchy of ecological organization (Basset, 1995; Petchey et al., 2008). Individuals’ energy demands are broadly dependent on body size (Gillooly et al., 2001; Kleiber, 1932; West et al., 1997) and closely related to space use features, for example, giving‐up time, density, and home range size (Börger et al., 2020; Brown, 1988; Ofstad et al., 2016). Therefore, differences in size may involve changing foraging strategies in order to meet energy needs, for example, ranging farther afield (larger home range) (Laver & Alexander, 2018) and giving up the patch earlier (shorter giving‐up time) and at higher densities of remaining resources (higher giving‐up density) when the absolute resource density remains constant (Basset & De Angelis, 2007; Brown et al., 1994; Cozzoli et al., 2020). The study of size dependency in individual space and resource use behavior can help to deepen our understanding of individual and population space and resource use, which, in turn, may enable prediction of future aquatic ecosystem functioning and resource availability.

The proposed case study involves an investigation of the size dependency of space and resource use behavior (in terms of giving‐up time and cumulative space used) and patch selection tendencies in macroinvertebrates. Motile macroinvertebrates are particularly challenging subjects for video tracking, because of their small juvenile size and translucent body. This experiment was conducted on small‐ to large‐sized males of *Gammarus insensibilis*.

## 2 MATERIALS AND METHODS

### 2.1 Model organism

*Gammarus insensibilis* (Stock, 1966) is an Atlantic‐Mediterranean amphipod species living in transitional and coastal waters (Costello, Emblow & White, 2001). Species of the genus *Gammarus* are ecologically highly successful due to their broad trophic repertoire; foraging flexibility; migration ability; tendency to drift, which allows them to easily invade and colonize ecosystems; high reproductive capacity, with several broods per female per year and a high number of offspring; and relative longevity (Gerhardt et al., 2011; Shadrin et al., 2020). Gammarids are important components of aquatic ecosystem trophic webs, feeding on detritus and providing nourishment for secondary consumers (Costantini et al., 2014; Shokri et al., 2019). They feed on a wide variety of plants, preferably grazing microscopic fungi growing on submerged decaying plant material (Glazier, 2014; Nelson, 2011), with a daily consumption rate of 46%–103% of their body mass (Berezina, 2007). They are characterized by thigmotaxis (Kohler et al., 2018), that is, they seek to remain in contact with either a wall or a plant leaf.

### 2.2 Specimen collection and sorting

*Gammarus insensibilis* individuals were collected from the Cesine coastal lagoon in South–East Italy (40.218N, 18.238E) and transferred to the Biodiversity and Ecosystem Functioning Laboratory (*BIO4IU*) at the University of the Salento in thermo‐insulated containers filled with water from the sampling sites and aerated during transport. Authorization for the specimens’ collection was issued by the competent authority (*World Wildlife Fund for Nature, Italy*). The species involved in this study are not endangered or protected. Specimens were maintained in the laboratory's aquaria for two weeks at a temperature of 18 °C and a salinity of 7 PSU, similar to the field sampling site. Decaying reed leaves were supplied as food in the aquaria and renewed depending on consumption. Before the start of the experiment, specimens were sorted by sex under a Nikon stereoscope (SMZ1270). Only males were selected for laboratory experiments, since oocyte production in females may induce nonsize‐related variability in energy requirements and individual space use behavior (Glazier et al., 2011). The experiment was performed in a controlled‐climate environment at a temperature of 18 ± 0.3°C and a salinity of 7 PSU. After every experimental trial, the animals were dried individually in an oven at 60°C for 72 hr and weighed to the nearest ± 0.001 mg.

### 2.3 Trophic resource conditioning

Leaves of *Phragmites australis* (Cav.) Trin. ex Steud were collected at the site of the specimens’ collection in early spring, cut into approximately 15 cm lengths, dried in the oven at 60 °C for 72 hr, weighed into separate portions (1 g and 0.5 g DW), and placed in 5 mm mesh plastic bags. The leaves were then leached and conditioned for two weeks in running environmental water at 18°C. The nutritional quality of the leaves is known to increase during conditioning because of microbial colonization and the assimilation of nutrients from the water by fungi and bacteria (Boling et al., 1975; Marks, 2019).

### 2.4 Experimental setup

**Figure 1:**
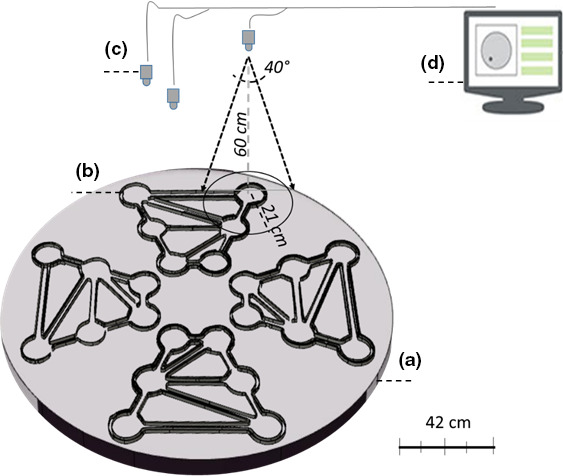
Diagram of the study setup: (a) Near infrared backlight source; (b) The maze, consisting of four blocks, each consisting of six patches; (c) Three cameras for each block; (d) Computer with an installed media recorder and Ethovision X14 software

**Figure 2:**
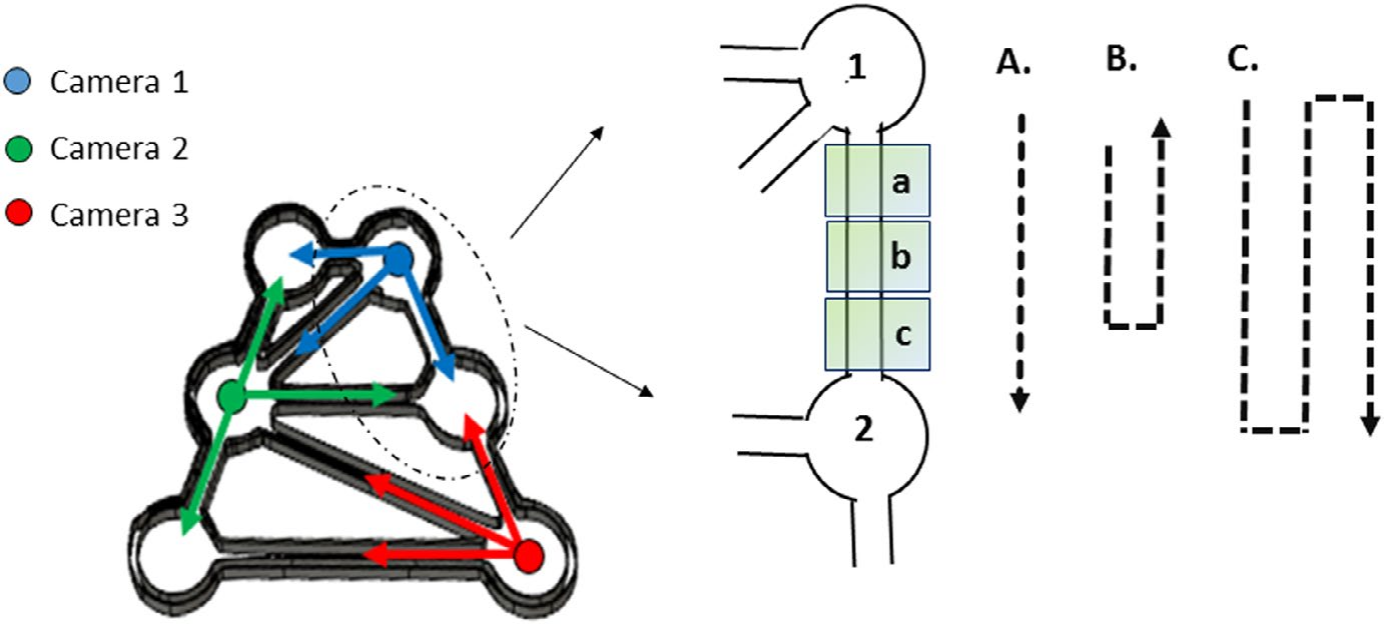
Left side: The positions of the cameras above the maze, making it possible to capture everything occurring inside the patches and channels. Each color represents a camera, and the arrows show its field of view. Right side: Schematic of two patches and the threshold between them (consisting of three subzones: a, b, and c) used for detecting the animal and counting the transitions. A, B, and C show plausible directional movements of an individual between the patches. A = the animal passes through the channel from patch 1 to patch 2 (crossing abc subzones; transition counted as + 1). B = the animal goes through part of the channel and then returns (crossing ab subzones; transition counted as 0). C = the animal passes through the channel from patch 1 to patch 2, returns to patch 1 and again passes through the channel from patch 1 to patch 2, in less than 30 s (crossing abc, cba, abc subzones; transition counted as + 1, −1, +1)

The experimental system consisted of a number of mazes made of transparent Plexiglas and designed specifically for measuring aquatic macroinvertebrate space use behavior, which were placed on top of a near infrared (*NIR*) backlight source. Each maze was in the shape of an isosceles trapezoid [with the parallel sides measuring 70 and 40 cm and each nonparallel side measuring 70 cm], inside which were six circular patches [13 cm in diameter, 3 cm high], each with an area of 0.013 m^2^, connected by a network of channels [2.5 cm wide, 3 cm high], with a total surface area of 0.08 m^2^ (Figure 1). For each experimental trial, 1 g DW of conditioned leaf fragments was uniformly distributed across the surface of one patch, 0.5 g of conditioned leaf fragments was placed in another patch (thereby simulating two resource patches), and the other four patches were left empty. The distribution of the resource patches was randomized in each test to prevent any effect of microcosm geometry. Individual movement in the experimental maze was recorded by cameras (Basler, aca1300‐60gm) equipped with infrared pass filters (850 E, 35.5 mm; Heliopan, Germany). The cameras were supported by an aluminum framework and positioned 60 cm above the maze with an aerial view of the microcosm and channels in order to detect individual movements (Figure 1). Because all the channels are in a radial position with respect to the center of the patches, placing the camera above the center of a patch ensured that it could capture everything occurring inside the patch and the channels connected to it. The setup of the cameras was crucial to the method, since the small size of the aquatic invertebrates is compounded by their tendency to thigmotaxis, which causes them to crawl or swim along the walls and would make it easy to miss an animal if a wall were blocking the view of the cameras. To this end, three cameras were placed at 60 cm above each maze. Each camera covered a view of two patches and the channels connected to them [with a visual radius of 21 cm] (Figures 1 and 2).

### 2.5 Automated tracking

Space use was measured for one individual at a time in an experimental maze, meaning that it was possible to know the position of the animal at any moment. Recordings were initiated 10 min after the specimen was placed in the maze and lasted for 6 hr. Videos were recorded at 25 frames per second with 1,280 × 1,024 spatial resolution. The video files were then processed automatically by Ethovision XT 14 in batch acquisition mode (Noldus Information Technology BV) [see Noldus et al. (2001) for more details on Ethovision software]. Specimens were directly identified by the software as moving elements compared to the static background, thus allowing detection even if the specimen was not moving. Ethovision XT 14 makes it possible to set transition thresholds and thus determine each transition between patches (an illustrative video is publicly accessible at https://osf.io/7ezm5/). The channels were divided into three zones (*a*, *b,* and *c*) (Figure 2), with transition being counted once the animal passed all three zones along the channel [depending on the direction, the transition was counted as positive or negative] (Figure 2). The data were then compiled at 30‐s intervals (defined by the user) and exported as text files. The output of Ethovision XT 14 was then fed into a Microsoft Excel Macro^©^™ which computed the time spent in each patch during each visit, as well as the number of patches visited, a visit being defined as each time an individual passes through a channel and enters a patch.

### 2.6 Data analysis

#### 2.6.1 System variables

The detection system has a large number of measurable parameters, such as distance moved, turning angle, angular speed, fractal dimension, and more (see https://www.noldus.com/ethovision‐xt/benefits). In terms of locomotory parameters, we mainly focused on mean speed (calculated as the mean distance travelled per time unit), acceleration (calculated as the rate of speed change per time unit), and step length (calculated as the mean distance travelled between two consecutive locations, (*x_t_*_‐1_ *, y_t_*_‐1_) and (*x_t,_ y_t_*), per time interval). The presence of the patch walls is expected to influence the individuals’ locomotory parameters (Cloyed & Dell, 2019; Uiterwaal et al., 2019). Thus, tracking information on individuals’ locomotory behavior from the outer 1 cm of the patch was excluded, leaving an arena 11 cm in diameter.

In terms of space use behavior, the present case study made use of four descriptors:

- The number of visits to each patch; a visit being defined as each time the animal entered a patch and remained in the patch for at least 30 s.
- The giving‐up time (*GUT*), defined as the duration of a single visit (Krebs et al., 1974), computed here as the average time spent in a resource patch during each visit.
- The total time spent by individuals in any patch in absolute terms (min) and as a fraction (%) of the experimental time.
- The individual cumulative space used, defined as the total area of the resource patches used by individuals during the experimental time (ca. 360 min), and it was computed simply as the number of visits to a resource patch × the resource patch area (0.01 m^2^).

In order to identify differences between the number of patches visited by fed and unfed individuals, we performed a nonparametric Kruskal–Wallis test. Since total time spent and average giving‐up time were not normally distributed, the impact of body weight and resource density on these two variables was tested with a Sheirer Ray Hare test. An ANCOVA was used to test the response of cumulative space used, total time spent in resource patches and average giving‐up time to body weight as an explanatory variable. The percentage of experimental period that animal spent in any patch was modeled by generalized (logistic) linear regression. The analyses were performed in the R free software environment (R Core Team 2019) using the *reshape* (Wickham, 2007), *lme4* (Bates et al., 2015), *sjPlot* (Lüdecke, 2018), and *traj* (McLean & Skowron Volponi, 2018) packages.

#### 2.6.2 Preliminary experimental setup

As part of the preliminary experimental setup, we tested conditions that could potentially affect the space use behavior of the model organism, namely:

- The internal ventilation system regulating room temperature.
- The near infrared backlight source (*NIR*).
- Foragers’ satiety levels.

The tests indicated that the ventilation system should be set at the lowest possible speed, as it could cause waves on the water surface and create noise in the detection of the animals. Another factor we tested was the effect of the near infrared backlight source (*NIR*) on forager behavior. We observed that the use of *NIR* had no influence on the number of patches visited or other primary locomotory behaviors (such as speed, acceleration, and step length). In addition, we tested the potential influence of the foragers’ satiety on space use behavior by comparing two groups of animals consisting of (a) unfed animals starved for 24 hr before the measurements and (b) fed animals taken directly from the aquaria. The exploratory behavior of unfed individuals was found to be less chaotic and more uniform, with low variation at the individual level over time (see “Fig. S1” in supporting information for more details). Therefore, we used unfed animals for the experiment (see the following chapter for detailed analysis).

## 3 RESULTS

### 3.1 Primary data

The specimens for which the system provided good data ranged in body length from 5.2 to 17.47 mm (12.08 [± 4.17] mm on average) and from 0.6 to 12.41 mg DW (6.9 [± 4.03] mg on average). The largest size corresponds to the maximum adult size sampled in nature. 15% of the animals used were found to be below the system detection limit: Since very small *G. insensibilis* specimens have translucent bodies, they do not generate enough contrast with the static background to be detected. Within the investigated size range, ca. 85% of the experimental trials successfully yielded a complete measurement.

**Figure 3:**
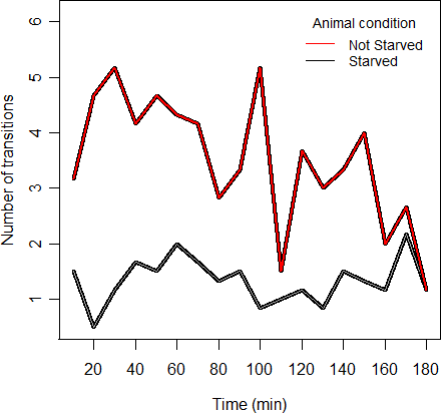
The average number of transitions in two groups of animals, starved and not starved, during 180 min; data compiled at 10‐min time intervals

The preliminary results based on two groups of animals, fed and unfed, showed that the coefficient of variation of patch visits among fed individuals was 62%, significantly higher than unfed individuals, among which it was 20% (Kruskal–Wallis; χ^2^ = 205.1, *p* < .05) (Figure 3).

#### 3.1.1 Locomotory data

**Figure 4:**
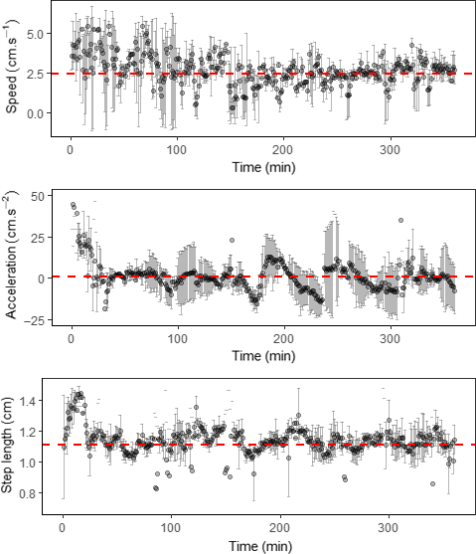
Individuals’ average (±*SD*) locomotory parameters: speed, acceleration, and step length during the experimental period. The dashed red lines show the average locomotory parameter values over time

Once the specimens were released into the experimental microcosm, they started actively exploring the mazes, with high speed and acceleration and a long step length for about the first 20 min, regardless of the amount of resources. However, the specimens then had lower speed and acceleration and a shorter step length, with little variation for the rest of the experimental time (Figure 4). The specimens had an average speed of 2.45 cm/s [± 1.03 *SD*], a maximum acceleration of 78.15 cm/s^2^, and an average step length of 1.11 cm [±0.15 *SD*] over the experimental time (ca. 360 min).

### 3.2 Space and resource use data

On average, individual *GUT* was 27.8 min [± 26.20 *SD*] and the cumulative space used over the experimental time (ca. 360 min) was 1.08 m^2^ [± 0.73 *SD*].

Body size and overall resource density in the patch both had a significant influence on individual *GUT* (respectively: Sheirer Ray Hare H = 3.14, *p* < .05; H = 59.5, *p* < .001). Furthermore, the influence of body size (Sheirer Ray Hare; H = 8.33, *p* < .01) and overall resource density (Sheirer Ray Hare; H = 56.8, *p* < .001) on the individual total time spent was also highlighted.

**Figure 5:**
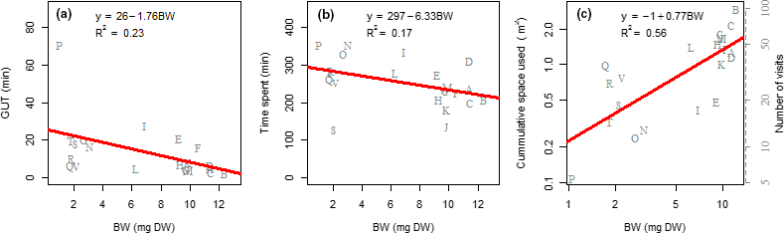
Linear relationships between the descriptors of individual space use and individual body weight (mg DW). Each letter on the scatter plot represents an individual. (a) average giving‐up time (min) in resources patches. (b) total time spent (min) in resources patches. (c) cumulative space used (m^2^), the secondary y axis showing the number of visits to the resource patch

Individual total time spent in resource patches (ANCOVA; F_1,19_ = 3.83, *p* < .05) and *GUT* (ANCOVA; F_1,19_ = 5.76, *p* < .05) decreased significantly with body size (Figure 5). However, individual cumulative space used increased with size, with a scaling coefficient of 0.77 (ANCOVA; F_1,19_ = 24.49, *p* < .05). The positive relationship with body size explained 56% of the variation in cumulative space used. In addition to cumulative space used, the number of visits scaled with individual body size, following the same trend.

**Figure 6:**
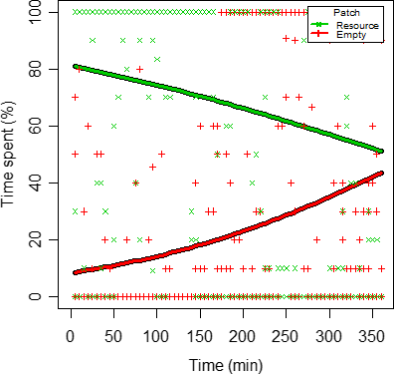
Individual time spent in resource patches (green) and empty patches (red) as a percentage of the total experimental time (ca. 360 min), modeled via logistic regression

Overall, individuals spent more than 70% of the first 150 min in resource patches (Figure 6). After this time, the percentage of time spent in resource patches dropped significantly, with a corresponding increase in the percentage of time spent in empty patches. To summarize, individuals showed foraging behavior at the beginning and shifted to explorative behavior over time (LR; *F* = 15.62, *p* < .05; Table 1).

**Table 1:** Relationship between individual time spent in empty and resource patches during the experimental time (ca. 360 min), modeled via logistic regression

Predictor	Odds ratio	CI	*p*
(Intercept)	0.26	0.20–0.33	<.001
Time	1.00	1.00–1.00	<.001
Resource	15.08	10.76–21.26	<.001
Time*Resource	0.99	0.99–1.00	<.001
Observation	2,880		
*R* ^2^ Tjur	.07		

## 4 DISCUSSION

Manipulative tests on the various factors influencing the spatial movement of aquatic invertebrates are still relatively rare (*some examples are* “Mancinelli (2010); Barnes et al. (2015); Kölzsch et al. (2015); Cloyed and Dell (2019); Cloyed et al. (2019)”). The results obtained in this study show that the adopted methodology offers a reliable approach to the systematic testing of hypotheses concerning the use of space and resources as well as patch selection, patch exploitation, and giving‐up time among small macroinvertebrates.

### 4.1 Methodology

The combined use of a near infrared (*NIR*) backlight source and the Ethovision X14 software proved to be an efficient way for observing aquatic macroinvertebrates while avoiding light reflections on the water surface that can interfere with the image analysis. One of the major advantages of the high contrast offered by *NIR* backlight is that it makes it possible to detect animals without marking them. Indeed, it is widely acknowledged that marking and tagging procedures lead to deviations from aquatic organisms’ normal behavior, altering locomotion parameters (Jones et al., 2013), activity levels and swimming performance (McCleave & Stred, 1975), reducing feeding and growth (Sutton et al., 2004) and compromising survival (Cottrill et al., 2006). For instance, Augusiak and Van den Brink (2015) observed that marking procedures significantly affected the turning angle, step length, and resting time of *Gammarus pulex*. Additionally, using *NIR* is appropriate for this kind of experiment because other light wavelengths could affect the behavior of aquatic invertebrates (Frank & Widder, 1994).

Using unfed individuals in the preliminary measurements reduced the randomness of the individuals’ movement and variations in their space use behavior at the population level. Since satiety and the potential degree of hunger in the aquaria vary among individuals, the use of unfed animals (starved for 24 hr before measurement) ensures physiological and metabolic equilibrium among individuals (Glazier et al., 2011), which is expected to reduce variation over time in their space use and foraging behavior.

We intentionally tested the detection setup on a very challenging (small, translucent, and fast‐moving) model organism to ensure the reliability and applicability of the method to other types of benthic invertebrate. The main limitations of the proposed methodology are its inability to track multiple individuals and long data‐acquisition times (about 12 hr).

### 4.2 Case study

To give a practical example of the potential of this method, we conducted a case study of space and resource use behavior among *G. insensibilis* individuals with various quantitative assessments including primary locomotory behavior, giving‐up time (*GUT*), and cumulative space used by individuals in resource patches within the experimental maze and time window (ca. 360 min).

Our observations regarding *G. insensibilis* locomotory parameters, that is, speed, acceleration, and step length, are generally consistent with earlier studies. The recent study by Augusiak and Van den Brink (2015) on the locomotory parameters of *Gammarus pulex* found an average step length of 1.31 cm [± 1.47 *SD*], a turning angle of 34.29° [± 88.8 *SD*], and a resting time of 39.5% [± 33.7 *SD*]. In line with these findings, (Longo et al., 2016) observed that *Gammarus aequicauda* had an average speed of 1.7 cm. s^‐1^ and a fractal dimension of 1.2.

Regarding space use behavior, our findings demonstrate that the cumulative space used positively scaled with individual body size with an allometric coefficient of 0.77. At the larger scale, McNab (1963) and Minns (1995) found that home range positively scaled with body size, with an exponent of 0.75. Furthermore, giving‐up time was observed to be characterized by negative size dependency in our setup. Thus, larger individuals gave up the resource patch earlier and travelled further in search of food than smaller ones. Similarly, recent studies have observed the negative size dependency of giving‐up time among several Gastropod species (Cozzoli et al., 2018, 2020).

The observed patterns of both individual *GUT* and cumulative space used in relation to individual body size are consistent with theory that predicts energy requirements increase with individual size (Gillooly et al., 2001; West et al., 1997). Morphological characteristics may also contribute to the locomotory efficiency of larger individuals, allowing them to have greater routine speed and a higher ability to discover new resource patches (Innes & Houlihan, 1985; Scales & Butler, 2016).

The method presented in this paper provides detailed information on the movement patterns of individuals and the various factors influencing them. The data obtained using this method can help to quantify species coexistence and yield insights into mechanistic models of space use on larger spatial scales.

## CONFLICT OF INTEREST

The authors declare that they have no conflict of interest.

## AUTHOR CONTRIBUTION

Milad Shokri: Conceptualization (lead); Data curation (lead); Formal analysis (lead); Investigation (lead); Methodology (lead); Software (lead); Validation (equal); Visualization (lead); Writing‐original draft (lead); Writing‐review & editing (lead). Francesco Cozzoli: Conceptualization (lead); Data curation (supporting); Formal analysis (supporting); Investigation (supporting); Methodology (supporting); Supervision (supporting); Writing‐review & editing (equal). Mario Ciotti: Visualization (supporting). Vojsava Gjoni: Visualization (supporting). Vanessa Marrocco: Visualization (supporting). Fabio Vignes: Visualization (supporting). Alberto Basset: Conceptualization (lead); Funding acquisition (lead); Methodology (equal); Supervision (lead); Validation (lead); Writing‐review & editing (equal).

## Supporting information

Figure S1

## Data Availability

Data are publicly accessible at the LifeWatch Italy Data Portal https://doi.org/10.48372/5T7Q‐0X08.

## References

[ece37129-bib-0001] Auger‐Méthé, M. , Derocher, A. E. , Plank, M. J. , Codling, E. A. , & Lewis, M. A. (2015). Differentiating the Lévy walk from a composite correlated random walk. Methods in Ecology and Evolution, 6, 1179–1189. 10.1111/2041-210X.12412

[ece37129-bib-0002] Augusiak, J. , & Van den Brink, P. J. (2015). Studying the movement behavior of benthic macroinvertebrates with automated video tracking. Ecology and Evolution, 5, 1563–1575. 10.1002/ece3.1425 25937901 PMC4409406

[ece37129-bib-0003] Avgar, T. , Baker, J. A. , Brown, G. S. , Hagens, J. S. , Kittle, A. M. , Mallon, E. E. , McGreer, M. T. , Mosser, A. , Newmaster, S. G. , Patterson, B. R. , Reid, D. E. B. , Rodgers, A. R. , Shuter, J. , Street, G. M. , Thompson, I. , Turetsky, M. J. , Wiebe, P. A. , & Fryxell, J. M. (2015). Space‐use behaviour of woodland caribou based on a cognitive movement model. Journal of Animal Ecology, 84, 1059–1070. 10.1111/1365-2656.12357 25714592

[ece37129-bib-0004] Barela, I. , Burger, L. M. , Taylor, J. , Evans, K. O. , Ogawa, R. , McClintic, L. , & Wang, G. (2020). Relationships between survival and habitat suitability of semi‐aquatic mammals. Ecology and Evolution, 10, 4867–4875. 10.1002/ece3.6239 32551067 PMC7297760

[ece37129-bib-0005] Barnes, A. D. , Spey, I.‐K. , Rohde, L. , Brose, U. , & Dell, A. I. (2015). Individual behaviour mediates effects of warming on movement across a fragmented landscape. Functional Ecology, 29, 1543–1552. 10.1111/1365-2435.12474

[ece37129-bib-0006] Basset, A. (1995). Body size‐related coexistence: an approach through allometric constraints on home‐range use. Ecology, 76, 1027–1035. 10.2307/1940913

[ece37129-bib-0007] Basset, A. , & De Angelis, D. L. (2007). Body size mediated coexistence of consumers competing for resources in space. Oikos, 116, 1363–1377. 10.1111/j.0030-1299.2007.15702.x

[ece37129-bib-0008] Bates, D. , Mächler, M. , Bolker, B. , & Walker, S. (2015). Fitting Linear Mixed‐Effects Models Using lme4. Journal of Statistical Software, 1(1), 2015.

[ece37129-bib-0009] Beckerman, A. , Petchey, O. L. , & Morin, P. J. (2010). Adaptive foragers and community ecology: Linking individuals to communities and ecosystems. Functional Ecology, 24, 1–6. 10.1111/j.1365-2435.2009.01673.x

[ece37129-bib-0010] Berezina, N. (2007). Food spectra and consumption rates of four amphipod species from the North‐West of Russia. Fundamental and Applied Limnology / Archiv Für Hydrobiologie, 168, 317–326. 10.1127/1863-9135/2007/0168-0317

[ece37129-bib-0011] Bogdziewicz, M. , Zwolak, R. , Redosh, L. , Rychlik, L. , & Crone, E. E. (2016). Negative effects of density on space use of small mammals differ with the phase of the masting‐induced population cycle. Ecology and Evolution, 6, 8423–8430. 10.1002/ece3.2513 28031794 PMC5167038

[ece37129-bib-0012] Boling, R. H. , Goodman, E. D. , Van Sickle, J. A. , Zimmer, J. O. , Cummings, K. W. , Petersen, R. C. , & Reice, S. R. (1975). Toward a model of detritus processing in a woodland stream. Ecology, 56, 141–151. 10.2307/1935306

[ece37129-bib-0013] Börger, L. , Fieberg, J. , Horne, J. S. , Rachlow, J. , Calabrese, J. M. , & Fleming, C. H. (2020). Animal home ranges: Concepts, uses, and estimation. In D. L. Murray , & B. K. Sandercock (Eds.), Population ecology in practice (pp. 315–332). John Wiley & Sons.

[ece37129-bib-0014] Brodersen, J. , Nilsson, P. A. , Hansson, L.‐A. , Skov, C. , & Brönmark, C. (2008). Condition‐dependent individual decision‐making determines cyprinid partial migration. Ecology, 89, 1195–1200. 10.1890/07-1318.1 18543613

[ece37129-bib-0015] Brown, J. S. (1988). Patch use as an indicator of habitat preference, predation risk, and competition. Behavioral Ecology and Sociobiology, 22, 37–47. 10.1007/BF00395696

[ece37129-bib-0016] Brown, J. S. , Kotler, B. P. , & Mitchell, W. A. (1994). Foraging theory, patch use, and the structure of a negev desert granivore community. Ecology, 75, 2286–2300. 10.2307/1940884

[ece37129-bib-0017] Cagnacci, F. , Focardi, S. , Heurich, M. , Stache, A. , Hewison, A. J. M. , Morellet, N. , Kjellander, P. , Linnell, J. D. C. , Mysterud, A. , Neteler, M. , Delucchi, L. , Ossi, F. , & Urbano, F. (2011). Partial migration in roe deer: Migratory and resident tactics are end points of a behavioural gradient determined by ecological factors. Oikos, 120, 1790–1802. 10.1111/j.1600-0706.2011.19441.x

[ece37129-bib-0018] Cayuela, H. , Pradel, R. , Joly, P. , & Besnard, A. (2017). Analysing movement behaviour and dynamic space‐use strategies among habitats using multi‐event capture‐recapture modelling. Methods in Ecology and Evolution, 8, 1124–1132. 10.1111/2041-210X.12717

[ece37129-bib-0019] Chakravarty, P. , Cozzi, G. , Ozgul, A. , & Aminian, K. (2019). A novel biomechanical approach for animal behaviour recognition using accelerometers. Methods in Ecology and Evolution, 10, 802–814. 10.1111/2041-210X.13172

[ece37129-bib-0020] Chapman, B. B. , Brönmark, C. , Nilsson, J.‐Å. , & Hansson, L.‐A. (2011). The ecology and evolution of partial migration. Oikos, 120, 1764–1775. 10.1111/j.1600-0706.2011.20131.x

[ece37129-bib-0021] Charnov, E. L. (1976). Optimal foraging, the marginal value theorem. Theoretical Population Biology, 9, 129–136. 1273796 10.1016/0040-5809(76)90040-x

[ece37129-bib-0022] Cloyed, C. S. , & Dell, A. I. (2019). Resource distribution and internal factors interact to govern movement of a freshwater snail. Proceedings of the Royal Society, 286, 20191610. 10.1098/rspb.2019.1610 PMC6784724 31551058

[ece37129-bib-0023] Cloyed, C. S. , Dell, A. I. , Hayes, T. , Kordas, R. L. , & O'Gorman, E. J. (2019). Long‐term exposure to higher temperature increases the thermal sensitivity of grazer metabolism and movement. Journal of Animal Ecology, 88, 833–844. 10.1111/1365-2656.12976 30873610

[ece37129-bib-0024] Cooke, S. J. , Woodley, C. M. , Brad Eppard, M. , Brown, R. S. , & Nielsen, J. L. (2011). Advancing the surgical implantation of electronic tags in fish: A gap analysis and research agenda based on a review of trends in intracoelomic tagging effects studies. Reviews in Fish Biology and Fisheries, 21, 127–151.

[ece37129-bib-0025] Costantini, M. L. , Calizza, E. , & Rossi, L. (2014). Stable isotope variation during fungal colonisation of leaf detritus in aquatic environments. Fungal Ecology, 11, 154–163.

[ece37129-bib-0026] Costello, M. J. , Emblow, C. , & White, R. J. (2001). European Register of Marine Species: A Check‐List of the Marine Species in Europe and a Bibliography of Guides to Their Identification. Collection Patrimoines naturels, Paris: Collection Patrimoines Naturels, Muséum national d'Histoire naturelle. https://books.google.it/books?id=58sTAQAAIAAJ

[ece37129-bib-0027] Cottrill, R. A. , Økland, F. , Aarestrup, K. , Jepsen, N. , Koed, A. , Hunter, K. J. , Butterworth, K. G. , & McKinley, R. S. (2006). Evaluation of three telemetry transmitter attachment methods for female silver‐phase American eels (*Anguilla rostrata Lesueur*). Journal of Great Lakes Research, 32, 502–511.

[ece37129-bib-0028] Cozzoli, F. , Ligetta, G. , Vignes, F. , & Basset, A. (2018). Revisiting GUD: An empirical test of the size‐dependency of patch departure behaviour. PLoS One, 13, e0204448. 30260989 10.1371/journal.pone.0204448 PMC6160073

[ece37129-bib-0029] Cozzoli, F. , Shokri, M. , Ligetta, G. , Ciotti, M. , Gjoni, V. , Marrocco, V. , Vignes, F. , & Basset, A. (2020). Relationship between individual metabolic rate and patch departure behaviour: Evidence from aquatic gastropods. Oikos, 129, 1657–1667.

[ece37129-bib-0030] Davidson, J. D. , & El Hady, A. (2019). Foraging as an evidence accumulation process. PLoS Computational Biology, 15(7), e1007060. 10.1371/journal.pcbi.1007060 31339878 PMC6682163

[ece37129-bib-0031] Davy‐Bowker, J. (2002). A mark and recapture study of water beetles (Coleoptera: Dytiscidae) in a group of semi‐permanent and temporary ponds. Aquatic Ecology, 36, 435–446.

[ece37129-bib-0032] de la Rosa, C. A. (2019). An inexpensive and open‐source method to study large terrestrial animal diet and behaviour using time‐lapse video and GPS. Methods in Ecology and Evolution, 10, 615–625. 10.1111/2041-210X.13146

[ece37129-bib-0033] Dell, A. I. , Bender, J. A. , Branson, K. , Couzin, I. D. , de Polavieja, G. G. , Noldus, L. P. J. J. , Pérez‐Escudero, A. , Perona, P. , Straw, A. D. , Wikelski, M. , & Brose, U. (2014). Automated image‐based tracking and its application in ecology. Trends in Ecology & Evolution, 29, 417–428. 10.1016/j.tree.2014.05.004 24908439

[ece37129-bib-0034] Edwards, A. M. , Phillips, R. A. , Watkins, N. W. , Freeman, M. P. , Murphy, E. J. , Afanasyev, V. , Buldyrev, S. V. , da Luz, M. G. , Raposo, E. P. , Stanley, H. E. , & Viswanathan, G. M. (2007). Revisiting Levy flight search patterns of wandering albatrosses, bumblebees and deer. Nature, 449, 1044–1048. 17960243 10.1038/nature06199

[ece37129-bib-0035] Fernandez, V. I. , Yawata, Y. , & Stocker, R. (2019). A foraging mandala for aquatic microorganisms. The ISME Journal, 13, 563–575. 10.1038/s41396-018-0309-4 30446738 PMC6461837

[ece37129-bib-0036] Frank, T. M. , & Widder, E. A. (1994). Evidence for behavioral sensitivity to near‐UV light in the deep‐sea crustacean Systellaspis debilis. Marine Biology, 118, 279–284. 10.1007/BF00349795

[ece37129-bib-0037] Froy, H. , Borger, L. , Regan, C. E. , Morris, A. , Morris, S. , Pilkington, J. G. , Crawley, M. J. , Clutton‐Brock, T. H. , Pemberton, J. M. , & Nussey, D. H. (2018). Declining home range area predicts reduced late‐life survival in two wild ungulate populations. Ecology Letters, 21, 1001–1009. 10.1111/ele.12965 29656580

[ece37129-bib-0038] Gerhardt, A. , Bloor, M. , & Mills, C. L. (2011). Gammarus: important taxon in freshwater and marine changing environments. International Journal of Zoology, 2011, 1–2. 10.1155/2011/524276

[ece37129-bib-0039] Getz, L. L. , Oli, M. K. , Hofmann, J. E. , & McGuire, B. (2005). Habitat‐specific demography of sympatric vole populations over 25 years. Journal of Mammalogy, 86, 561–568. 10.1644/1545‐1542(2005)86[561:HDOSVP]2.0.CO;2

[ece37129-bib-0040] Gillooly, J. F. , Brown, J. H. , West, G. B. , Savage, V. M. , & Charnov, E. L. (2001). Effects of size and temperature on metabolic rate. Science, 293, 2248–2251. 10.1126/science.1061967 11567137

[ece37129-bib-0041] Glazier, D. , Butler, E. M. , Lombardi, S. A. , Deptola, T. J. , Reese, A. J. , & Satterthwaite, E. V. (2011). Ecological effects on metabolic scaling: Amphipod responses to fish predators in freshwater springs. Ecological Monographs, 81, 599–618. 10.1890/11-0264.1

[ece37129-bib-0042] Glazier, S. D. (2014). Metabolic Scaling in Complex Living Systems. Systems, 2. 10.3390/systems2040451

[ece37129-bib-0043] Golet, W. J. , Scopel, D. A. , Cooper, A. B. , & Watson, W. H. III (2006). Daily patterns of locomotion expressed by American Lobsters (Homarus Americanus) in their natural habitat. Journal of Crustacean Biology, 26, 610–620. 10.1651/S-2729.1

[ece37129-bib-0044] Gurarie, E. , Andrews, R. D. , & Laidre, K. L. (2009). A novel method for identifying behavioural changes in animal movement data. Ecology Letters, 12, 395–408. 10.1111/j.1461-0248.2009.01293.x 19379134

[ece37129-bib-0045] Hagler, J. R. , & Jackson, C. G. (2001). Methods for marking insects: current techniques and future prospects. Annual Review of Entomology, 46, 511–543. 10.1146/annurev.ento.46.1.511 11112178

[ece37129-bib-0046] Holyoak, M. , Casagrandi, R. , Nathan, R. , Revilla, E. , & Spiegel, O. (2008). Trends and missing parts in the study of movement ecology. Proceedings of the National Academy of Sciences, 105, 19060. 10.1073/pnas.0800483105 PMC2614715 19060194

[ece37129-bib-0047] Humphries, N. E. , Weimerskirch, H. , Queiroz, N. , Southall, E. J. , & Sims, D. W. (2012). Foraging success of biological Lévy flights recorded in situ. Proceedings of the National Academy of Sciences, 109, 7169. 10.1073/pnas.1121201109 PMC3358854 22529349

[ece37129-bib-0048] Innes, A. J. , & Houlihan, D. F. (1985). Aerobic capacity and cost of locomotion of a cool temperate gastropod: A comparison with some Mediterranean species. Comparative Biochemistry and Physiology Part A: Physiology, 80, 487–493. 10.1016/0300-9629(85)90402-5

[ece37129-bib-0049] Jepsen, N. , Thorstad, E. B. , Havn, T. , & Lucas, M. C. (2015). The use of external electronic tags on fish: An evaluation of tag retention and tagging effects. Animal Biotelemetry, 3, 49. 10.1186/s40317-015-0086-z

[ece37129-bib-0050] Jones, T. , Houtan, K. S. V. , Bostrom, B. L. , Ostafichuk, P. , Mikkelsen, J. , Tezcan, E. , Carey, M. , Imlach, B. , & Seminoff, J. A. (2013). Calculating the ecological impacts of animal‐borne instruments on aquatic organisms. Methods in Ecology and Evolution, 4, 1178–1186.

[ece37129-bib-0051] Kleiber, M. (1932). Body size and metabolism. Hilgardia, 6, 315–353. 10.3733/hilg.v06n11p315

[ece37129-bib-0052] Kohler, S. A. , Parker, M. O. , & Ford, A. T. (2018). Shape and size of the arenas affect amphipod behaviours: Implications for ecotoxicology. PeerJ, 6, e5271. 10.7717/peerj.5271 30065877 PMC6064634

[ece37129-bib-0053] Kölzsch, A. , Alzate, A. , Bartumeus, F. , de Jager, M. , Weerman, E. J. , Hengeveld, G. M. , Naguib, M. , Nolet, B. A. , & van de Koppel, J. (2015). Experimental evidence for inherent Lévy search behaviour in foraging animals. Proceedings of the Royal Society B: Biological Sciences, 282, 20150424. 10.1098/rspb.2015.0424 PMC4424656 25904671

[ece37129-bib-0054] Koy, K. , & Plotnick, R. E. (2007). CHAPTER 25 ‐ Theoretical and experimental ichnology of mobile foraging. In W. Miller (Ed.), Trace fossils (pp. 428–441). Elsevier.

[ece37129-bib-0055] Krebs, J. R. , Ryan, J. C. , & Charnov, E. L. (1974). Hunting by expectation or optimal foraging? A study of patch use by chickadees. Animal Behavior, 22, 953‐IN953. 10.1016/0003-3472(74)90018-9

[ece37129-bib-0056] Lagerspetz, K. Y. H. , & Vainio, L. A. (2006). Thermal behaviour of crustaceans. Biological Reviews, 81, 237–258. 10.1017/S1464793105006998 16522227

[ece37129-bib-0057] Lameris, T. K. , & Kleyheeg, E. (2017). Reduction in adverse effects of tracking devices on waterfowl requires better measuring and reporting. Animal Biotelemetry, 5, 24. 10.1186/s40317-017-0139-6

[ece37129-bib-0058] Laver, P. N. , & Alexander, K. A. (2018). Association with humans and seasonality interact to reverse predictions for animal space use. Movement Ecology, 6, 5. 10.1186/s40462-018-0123-7 29736242 PMC5924504

[ece37129-bib-0059] Longo, E. , Verschut, T. , Carrozzo, L. , Zotti, M. , & Mancinelli, G. (2016). Inter‐ and intra‐specific variation in movement behaviour of benthic macroinvertebrates from a transitional habitat: A laboratory experiment. Rendiconti Lincei, 27, 281–290. 10.1007/s12210-015-0475-5

[ece37129-bib-0060] Lüdecke, D. (2018). sjmisc: Data and variable transformation functions. Journal of Open Source Software, 3, 754. 10.21105/joss.00754

[ece37129-bib-0061] Makin, D. F. , & Kotler, B. P. (2019). How do Allenby’s gerbils titrate risk and reward in response to different predators? Behavioral Ecology and Sociobiology, 74, 6.

[ece37129-bib-0062] Mancinelli, G. (2010). Intraspecific, size‐dependent variation in the movement behaviour of a brackish‐water isopod: A resource‐free laboratory experiment. Marine and Freshwater Behaviour and Physiology, 43, 321–337.

[ece37129-bib-0063] Marks, J. C. (2019). Revisiting the fates of dead leaves that fall into streams. Annual Review of Ecology, Evolution, and Systematics, 50, 547–568.

[ece37129-bib-0064] McCleave, J. D. , & Stred, K. A. (1975). Effect of dummy telemetry transmitters on stamina of Atlantic salmon (*Salmo salar*) smolts. Journal of the Fisheries Board of Canada, 32, 559–563.

[ece37129-bib-0065] McLean, D. J. , & Skowron Volponi, M. A. (2018). trajr: An R package for characterisation of animal trajectories. Ethology, 124, 440–448.

[ece37129-bib-0066] McNab, B. K. (1963). Bioenergetics and the determination of home range size. The American Naturalist, 97, 133–140.

[ece37129-bib-0067] Melbinger, A. , & Vergassola, M. (2015). The impact of environmental fluctuations on evolutionary fitness functions. Scientific Reports, 5, 15211. 26477392 10.1038/srep15211 PMC4609966

[ece37129-bib-0068] Minns, C. K. (1995). Allometry of home range size in lake and river fishes. Canadian Journal of Fisheries and Aquatic Sciences, 52, 1499–1508.

[ece37129-bib-0069] Mysterud, A. , Loe, L. E. , Zimmermann, B. , Bischof, R. , Veiberg, V. , & Meisingset, E. (2011). Partial migration in expanding red deer populations at northern latitudes – a role for density dependence? Oikos, 120, 1817–1825.

[ece37129-bib-0070] Nathan, R. , Getz, W. M. , Revilla, E. , Holyoak, M. , Kadmon, R. , Saltz, D. , & Smouse, P. E. (2008). A movement ecology paradigm for unifying organismal movement research. Proceedings of the National Academy of Sciences, 105, 19052. 10.1073/pnas.0800375105 PMC2614714 19060196

[ece37129-bib-0071] Nelson, D. (2011). Gammarus‐microbial interactions: A review. International Journal of Zoology, 2011, 1–6.

[ece37129-bib-0072] Noldus, L. P. , Spink, A. J. , & Tegelenbosch, R. A. (2001). EthoVision: A versatile video tracking system for automation of behavioral experiments. Behav Res Methods Instrum Comput, 33, 398–414. 10.3758/BF03195394 11591072

[ece37129-bib-0073] Nonacs, P. (2001). State dependent behavior and the Marginal Value Theorem. Behavioral Ecology, 12, 71–83. 10.1093/oxfordjournals.beheco.a000381

[ece37129-bib-0074] Ofstad, E. G. , Herfindal, I. , Solberg, E. J. , & Sæther, B.‐E. (2016). Home ranges, habitat and body mass: Simple correlates of home range size in ungulates. Proceedings of the Royal Society B: Biological Sciences, 283, 20161234. 10.1098/rspb.2016.1234 PMC5204158 28003441

[ece37129-bib-0075] Noldus Information Technology BV . Nieuwe Kanaal 5, 6709 PA, Wageningen, The Netherlands.

[ece37129-bib-0076] Petchey, O. L. , Beckerman, A. P. , Riede, J. O. , & Warren, P. H. (2008). Size, foraging, and food web structure. Proceedings of the National Academy of Sciences, 105, 4191. 10.1073/pnas.0710672105 PMC2393804 18337512

[ece37129-bib-0077] Post, D. M. , Palkovacs, E. P. , Schielke, E. G. , & Dodson, S. I. (2008). Intraspecific variation in a predator affects community structure and cascading trophic interactions. Ecology, 89, 2019–2032. 10.1890/07-1216.1 18705387

[ece37129-bib-0078] Potts, J. R. , Bastille‐Rousseau, G. , Murray, D. L. , Schaefer, J. A. , & Lewis, M. A. (2014). Predicting local and non‐local effects of resources on animal space use using a mechanistic step selection model. Methods in Ecology and Evolution, 5, 253–262. 10.1111/2041-210X.12150 25834721 PMC4375923

[ece37129-bib-0555] R Core Team (2019). R: A language and environment for statistical computing. Vienna, Austria: R Foundation for Statistical Computing.

[ece37129-bib-0079] Reubens, J. , Verhelst, P. , van der Knaap, I. , Wydooghe, B. , Milotic, T. , Deneudt, K. , Hernandez, F. , & Pauwels, I. (2019). The need for aquatic tracking networks: The Permanent Belgian Acoustic Receiver Network. Animal Biotelemetry, 7, 2. 10.1186/s40317-019-0164-8

[ece37129-bib-0080] Robinson, E. J. H. , Richardson, T. O. , Sendova‐Franks, A. B. , Feinerman, O. , & Franks, N. R. (2009). Radio tagging reveals the roles of corpulence, experience and social information in ant decision making. Behavioral Ecology and Sociobiology, 63, 627–636. 10.1007/s00265-008-0696-z

[ece37129-bib-0081] Roche, D. G. , Careau, V. , & Binning, S. A. (2016). Demystifying animal ‘personality’ (or not): Why individual variation matters to experimental biologists. The Journal of Experimental Biology, 219, 3832. 10.1242/jeb.146712 27852750

[ece37129-bib-0082] Scales, J. A. , & Butler, M. A. (2016). Adaptive evolution in locomotor performance: How selective pressures and functional relationships produce diversity. Evolution, 70, 48–61. 10.1111/evo.12825 26614565

[ece37129-bib-0083] Shadrin, N. , Yakovenko, V. , & Anufriieva, E. (2020). Gammarus aequicauda and Moina salina in the Crimean saline waters: New experimental and field data on their trophic relation. Aquaculture Research, 51, 3091–3099.

[ece37129-bib-0084] Shokri, M. , Ciotti, M. , Vignes, F. , Gjoni, V. , & Basset, A. (2019). Components of standard metabolic rate variability in three species of gammarids. Web Ecology, 19, 1–13.

[ece37129-bib-0085] Smith, E. A. , Bettinger, R. L. , Bishop, C. A. , Blundell, V. , Cashdan, E. , Casimir, M. J. , Christenson, A. L. , Cox, B. , Dyson‐Hudson, R. , Hayden, B. , Richerson, P. J. , Roth, E. A. , Simms, S. R. , & Stini, W. A. (1983). Anthropological applications of optimal foraging theory: A critical review [and comments and Reply]. Current Anthropology, 24, 625–651. 10.1086/203066

[ece37129-bib-0086] Stevens, J. (2010). The challenges of understanding animal minds. Frontiers in Psychology, 1, 203. 10.3389/fpsyg.2010.00203 21833259 PMC3153809

[ece37129-bib-0087] Stock, J. H. (1966). A key to the species of the locusta‐group of the amphipod genus Gammarus, with notes on their nomenclature. Bulletin Zoologisch Museum Amsterdam, 1–5.

[ece37129-bib-0088] Sutton, T. M. , Volkman, E. T. , Pangle, K. L. , Rajchel, D. A. , & Duehr, J. P. (2004). Effects of absorbable suture strand diameter on retention of external radio transmitters by juvenile lake sturgeon. North American Journal of Fisheries Management, 24, 1404–1408. 10.1577/M03-194.1

[ece37129-bib-0089] Uiterwaal, S. F. , Dell, A. I. , & DeLong, J. P. (2019). Arena size modulates functional responses via behavioral mechanisms. Behavioral Ecology, 30, 483–489. 10.1093/beheco/ary188

[ece37129-bib-0090] West, G. B. , Brown, J. H. , & Enquist, B. J. (1997). A general model for the origin of allometric scaling laws in biology. Science, 276, 122. 10.1126/science.276.5309.122 9082983

[ece37129-bib-0091] Wickham, H. (2007). Reshaping Data with the reshape Package. Journal of Statistical Software, 1(12), 2007.

