# Supplementary material for: A new approach to assessing the space use behavior of macroinvertebrates by automated video tracking
Source: Ecol Evol. 2021 Mar 13;11(7):3004–14. doi: 10.1002/ece3.7129 (PMC8019041; doi:10.1002/ece3.7129)
Supplement: Supplementary file 1 — Figure S1 [file ECE3-11-3004-s001.pdf]

Supporting Information

A new approach to assessing the space use behaviour of macroinvertebrates by automated video tracking

*Milad Shokri[1, *], Francesco Cozzoli[1,2], Mario Ciotti[1], Vojsava Gjoni[1], Vanessa Marrocco[1], Fabio Vignes[1], Alberto Basset[1]*

Appendix A1

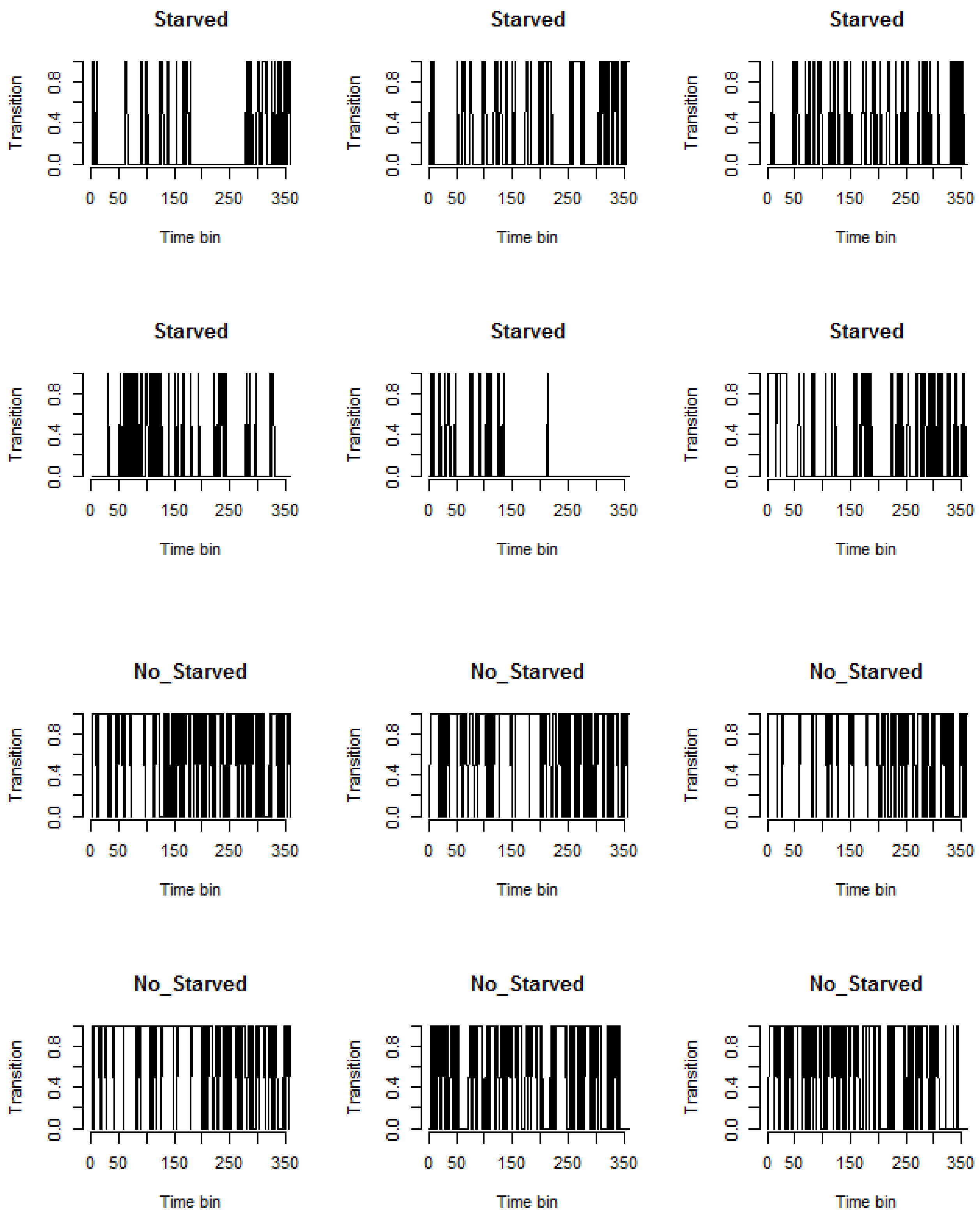

Figure A1. Transition between patches (1 = the animal passes the threshold which consists of three

subzones, and 0 = the animal remains stationary, or doesn’t cross entirely the threshold which consists of three subzones) in starved and non-starved groups of animals, the data compiled at 30 second intervals.
